# Supplementary material for: Self-reported dyspareunia and outcome satisfaction after spontaneous second-degree tear compared to episiotomy: A register-based cohort study
Source: PLoS One. 2024 Dec 19;19(12):e0315899. doi: 10.1371/journal.pone.0315899 (PMC11658579; doi:10.1371/journal.pone.0315899)
Supplement: Attachment — Submitted filename: Comments to the Author.docx [file pone.0315899.s001.docx]

Comments to the Author

-Unfortunately, in my opinion, this article hasn't any novelty and is one of the most repeated articles that have been studied from all aspects.

- There is no innovation, the results are predictable before the evaluation A study with a larger sample size showed that episiotomy causes dyspareunia. What is the gap of the study? The results are predictable before the review

- According to Table 1, two groups have significant differences in terms of labor and postpartum complications, which affects the results

-- The conclusion should be written as a statement, not repeating the results

- Grammar errors and languages. There are many grammar errors in the manuscript that require substantial improvement.
