# Supplementary material for: Self-reported dyspareunia and outcome satisfaction after spontaneous second-degree tear compared to episiotomy: A register-based cohort study
Source: PLoS One. 2024 Dec 19;19(12):e0315899. doi: 10.1371/journal.pone.0315899 (PMC11658579; doi:10.1371/journal.pone.0315899)
Supplement: Attachment — Submitted filename: Response to Reviewers.docx [file pone.0315899.s002.docx]

Uppsala 9^th^ of October 2024

Dear Editors,

Thank you very much for your time and effort with our manuscript. Your questions and comments have been helpful for us to make improvements and alterations to the text. Our answers and changes are listed in the table below.

| **Reviewer 1** | **Author’s reply** |
| --- | --- |
| In the summary, the year and the place of the study should be mentioned. | In the abstract, the year was already stated however the place (Sweden) is now added. |
| In the conclusion, a summary should be written “considering that..............................., it is suggested …. .... .... (write your suggestions for clinical practice and scientific research ) | Thank you for the suggestion. The conclusion is altered and the suggested sentenced is added (conclusion page 13). |
| In Table 3, bold the main variables to distinguish them from the sub-variables, such as : delivery mood, pain at 8 weeks, postpartum infection, etc., or use indentation. | In Table 3, as well as in table 4, the main variables have been changed to bold font. |
| Although breastfeeding causes vaginal dryness, it does not lead to reportable dyspareunia, however, please find more references for this issue, otherwise delete the sentence. | We agree and the sentence in the discussion (page 11, line 249) is changed and two more references have been added. |
| According to the very good study that has been done and the insight that the researchers have found on the subject, it is suggested to propose a questionnaire development study for designing a tool investigated the consequences of spontaneous and non-spontaneous vaginal tears so that it can be used by other researchers and also policymakers could be familiar with further variables that must be included into the registries. | Thank you for the comment. We suggest that the risk factors identified in our study can be used by other researchers and by health care providers. This is mentioned in the discussion (conclusion). Furthermore, we added a sentence on subclassification of second-degree tears, including levator ani injury, in the Swedish version of ICD-10 codes that has been added recently and can be used as a variable in future research (discussion last paragraph). |
| **Reviewer 2** |  |
| Unfortunately, in my opinion, this article hasn't any novelty and is one of the most repeated articles that have been studied from all aspects.  There is no innovation, the results are predictable before the evaluation A study with a larger sample size showed that episiotomy causes dyspareunia. What is the gap of the study? The results are predictable before the review | We regret that the reviewer finds that our study has shortcomings. We have rewritten the manuscript to emphasize that we study self-reported *degree of* dyspareunia and *level of* satisfaction. This was unclear in the earlier version.  In our knowledge, degree of dyspareunia and level of satisfaction after second-degree tear have not previously been studied. The large study population of more than 5000 women add strength to its findings. We are not aware of a larger study that looks at second-degree tear on its own. |
| According to Table 1, two groups have significant differences in terms of labor and postpartum complications, which affects the results | We are aware of the differences between the groups and we have tried to be transparent about the differences in the text and table 1. The post-partum complications are exposures that were adjusted for maternal characteristics and mode of delivery when we present the outcomes (table 3 and 4). |
| The conclusion should be written as a statement, not repeating the results | The conclusion has been altered so that it is now a statement instead of repeating the results. |
| Grammar errors and languages. There are many grammar errors in the manuscript that require substantial improvement. | The paper has been proof read. Grammatical and language errors have been corrected. |
| **Journal requirements** |  |
| Please ensure that your manuscript meets PLOS ONE's style requirements, including those for file naming. | Manuscript including tables, authors’ affiliation and headings updated according to PLOS ONE’s style requirements. Font size has been altered. |
| Please remove any funding-related text from the manuscript and let us know how you would like to update your Funding Statement. | The funding-related text has been removed. The Acknowledgements section now states: “The authors would like to thank all participants in the Perineal Laceration Registry who took their time to complete the questionnaires.” |
| Please note that your Data Availability Statement is currently missing the repository name and/or the DOI/accession number of each dataset OR a direct link to access each database. If your manuscript is accepted for publication, you will be asked to provide these details on a very short timeline. We therefore suggest that you provide this information now, though we will not hold up the peer review process if you are unable. | Unfortunately, register data cannot be made publicly available under Swedish privacy laws. The data for this research project has been exported from the National Board of Health and Welfare in Sweden, which does not permit data-sharing according to the Swedish Secrecy Act. However, investigators may apply to access the study data by contacting the Swedish National Board of Health and Welfare ([registerservice@socialstyrelsen.se](mailto:registerservice@socialstyrelsen.se)) |
| Please include a separate caption for each figure in your manuscript. | A caption for the figure has been added in the text (methods, page 5). |

Yours sincerely,

Maria Jonsson *M.D. Ph.D. Professor*

Mette L Josefsson *MBBS MRes MRCOG*

Department of Women’s and Children’s Health

Uppsala University, Uppsala, Sweden
